# Numerical Evaluation and Prediction of Porous Implant Design and Flow Performance

**DOI:** 10.1155/2018/1215021

**Published:** 2018-06-12

**Authors:** Jian Li, Diansheng Chen, Huiqin Luan, Yingying Zhang, Yubo Fan

**Affiliations:** ^1^Robotic Institute, Beihang University, Beijing 100191, China; ^2^Beijing Key Laboratory of Rehabilitation Technical Aids for Old-Age Disability and Key Laboratory of Rehabilitation Aids Technology and System of the Ministry of Civil Affairs, National Research Center for Rehabilitation Technical Aids, Beijing 100176, China; ^3^Beijing Advanced Innovation Center for Biomedical Engineering, Beihang University, Beijing 100191, China; ^4^Key Laboratory for Biomechanics and Mechanobiology of Ministry of Education, School of Biological Science and Medical Engineering, Beihang University, Beijing 100191, China

## Abstract

Porous structure has been widely acknowledged as important factor for mass transfer and tissue regeneration. This study investigates effect of aimed-control design on mass transfer and tissue regeneration of porous implant with regular unit cell. Two shapes of unit cells (Octet truss, and Rhombic dodecahedron) were selected, which have similar symmetrical structure and are commonly used in practice. Through parametric design, porous scaffolds with the strut sizes of *φ* 0.5, 0.7, 0.9, and 1.1mm were created, respectively. Then using fluid flow simulation method, flow velocity, permeability, and shear stress which could reflect the properties of mass transfer and tissue regeneration were compared and evaluated, and the relationships between porous structure's physical parameters and flow performance were studied. Results demonstrated that unit cell shape and strut size greatly determine and influence other physical parameters and flow performances of porous implant. With the increasing of strut size, pore size and porosity linearly decrease, but the volume, surface area, and specific surface area increased. Importantly, implant with smaller strut size resulted in smaller flow velocity directly but greater permeability and more appropriate shear stress, which should be beneficial to cell attachment and proliferation. This study confirmed that porous implant with different unit cell shows different performances of mass transfer and tissue regeneration, and unit cell shape and strut size play vital roles in the control design. These findings could facilitate the quantitative assessment and optimization of the porous implant.

## 1. Introduction

Porous structure has been widely acknowledged as important factor to avoid stress shielding and promote mass transfer, cell adhesion, and differentiation for bone tissue engineering (BTE), which could be manufactured by conventional fabrication techniques [1], such as gas foaming, solvent casting, particle leaching, fiber meshes, and freeze drying [2]. However, these methods should lead to irregular porous structure and uncontrollable interconnectivity, which have many flaws and potential risks for mechanical properties and biological properties, such as stress concentration and fatigue damage. Additive manufacturing (AM), also commonly known as 3D printing, is a process of joining materials layer by layer [3], provides required ability to deliver a high level of control over the complex architecture of the construct, and has been found to be advantageous for BTE. With the advent of AM [4], structure with different unit cells could be tailored in the design, which has great significance for the porous implant [5].

In general, ideal implants for BTE are expected to provide sufficient mechanical strength and stimulate cell attachment, viability, and proliferation so as to implant fixation to the host bone and tissue regeneration [6]. It has been reported in the literatures that physical parameters of porous structure, such as pore size, porosity, volume, surface area, and specific surface area, could affect mass transfer and cell differentiation. Roman A. Perez et al. reviewed that the pore size and pore size distribution as well as the pore morphology are key parameters that play a critical role in balancing the physical and biological properties [7]. Jie Fan et al. found that larger scaffold pore size has been shown to enhance osteoid tissue ingrowth and greater porosity was beneficial to proliferation of seeded cells [8]. Natalja E. Fedorovich, M.D., et al. proposed that the porosity is important for cell/tissue conductive properties [9]. Ju-Ang Kim et al. proved that high porosity promoted rapid biodegradation and bone regeneration for scaffolds [10]. A. T. Sidambe et al. summarized that porous structures with rougher surfaces were slightly more compatible for cell attachment and proliferation due to larger specific surface area than the smoother surface [11]. Meanwhile, suitable permeability and mechanical stimuli (shear stress) inside the porous implant also has important influence on cell proliferation and differentiation [12, 13]. Jie Fan et al. demonstrated that higher permeable scaffolds exhibited superior performance during bone regeneration in vitro and the advantages of higher scaffold permeability were amplified in perfused culture [8]. Karande TS suggests that permeability characterized the ability of nutrient delivery, waste removal, and cell migration [14]. Anna G. Mitsak et al. found that permeability increased with higher pore volume and resulted in better bone regeneration and blood vessel infiltration when other pore parameters were kept the same [15]. Porter et al. investigated the flow in scaffolds and found an average shear stress of 0.05 mPa was required to have stimulating effect on cell proliferation and that higher shear stress would lead to subsequent upregulation of osteoblast growth [16]. Cartmell et al. suggested that, for a positive effect on seeded cell viability and proliferation in vitro, fluid shear stress ranging from 0.05 to 25 mPa was desired [17]. Raimondi et al. perfused and predicted that a wall shear stress in the range 1.5–13.5 mPa was required for the stimulation of higher cell viability [18, 19]. It appears that although many studies had involved some rules for pore size, porosity, and permeability [7–10], the design of the porous structure is currently based on simple empirical level, such as minimum pore and interconnects sizes, and direct determinant factors for physical parameters of porous structure were unclear. In the same line of thought, two clinical rationales for the porous implant should be stressed [20–22]. Firstly, the implant should match the mechanical properties of the host bone in coping with load transfers and prosthesis fixation [23]. Secondly, the implant should provide better surroundings for mass transfer and tissue regeneration [24, 25]. Importantly, in practice, unit cell selection is a key step to “fit” the implant with appropriate mechanical properties and biological performance in a typical design [26]. However, currently in literature, different kinds of pores are selected just by engineers' experience in clinic, and quantitative, intuitive, and controllable design of biological performance for porous implant is urgently lacking [27]. Therefore, how to design an ideal porous implant purposefully and directly is still a key problem.

In addition, there are four methods for the porous structure design [28]: (1) computer assisted design (CAD), for example, SolidWorks software (Dassault Systèmes, Velizy-Villacoublay, France) could be used for the parameter design, and Boolean operations including union, subtraction and intersection would give great help for the complex structure's creation; (2) Implicit Surface (IS) modeling of mathematic trigonometric functions is used to maximize the surface-volume ratio of the implant; (3) microcomputed tomography (*μ*CT) and image data of original bone have been used as the basis for the design; (4) topology optimization and the best shape could be got by software simulation when the corresponding material, force, and fixed boundary conditions were given to the model. Although none of the above methods exhibit overwhelming superiority, CAD method has more controllability, modifiability, and intuitiveness and is very suited to control design for the porous implant. Moreover, using CAD method, almost all of the physical parameters (pore size, porosity, volume, surface area, permeability, and interconnectivity) could be tailored and adjusted by the right unit cell shape and strut size [26, 28], but previous researches seemed to ignore strut size, whilst quantitative relationships between strut size and other physical parameters, permeability, mechanical stimuli, and biological responses are still not well known.

Herein, this study focused on the CAD design and investigated the effect of control design on mass transfer and tissue regeneration of porous implant with regular unit cell. Two shapes of unit cell were selected and corresponding scaffolds with different strut sizes (*φ*0.5, 0.7, 0.9, and 1.1mm) were created. Using computational flow simulation methodology, predictions of design parameters, especially effects of strut size on flow velocity, permeability, and shear stress were compared and evaluated, which would result in different biological properties for porous implant.

## 2. Methods

### 2.1. Control Design of Porous Scaffolds

**Figure 1:**
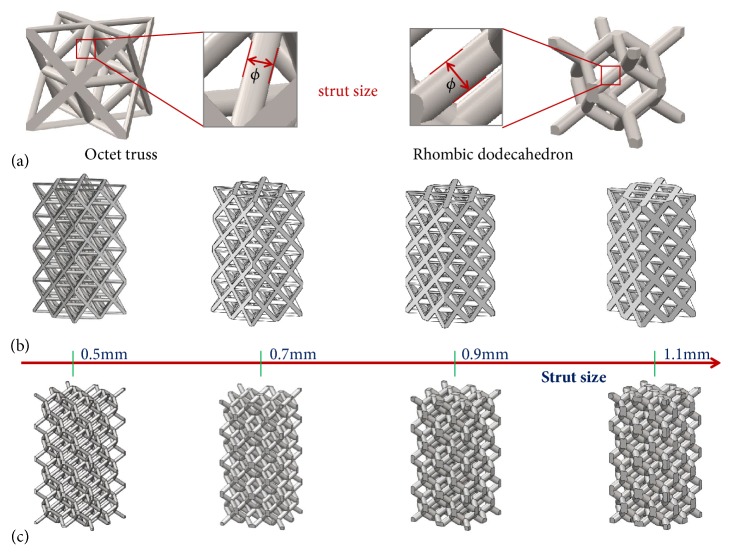
Regular unit cells and related scaffolds design: (a) two shapes of unit cells; (b) OT scaffolds; (b) RD scaffolds.

In this study, regular unit cells, Octet truss (OT) [29], and Rhombic dodecahedron (OT) [30] in the Magics software (Materialise, Leuven, Belgium) were selected (Figure 1(a)), which have similar symmetrical structure both on the coronal plane and on the sagittal plane, and are commonly used in practice. In the SolidWorks software (Dassault Systèmes, Velizy-Villacoublay, France), parametric models refer to single unit cell designed with dimensions 5 mm x 5 mm x 5 mm for OT and RD firstly [31]. Further on, using mirror modeling operation, single unit cells were repeated along* x*-,* y*-, and* z*-axis periodically in the software; porous scaffolds, 10 mm in length and width, and 20 mm in height, with the strut size of *φ*0.5, 0.7, 0.9, and 1.1 were created (Figures 1(a) and 1(b)), respectively, which had 4 layers and 16 complete single pores, and are entitled by OT-0.5, OT-0.7, OT-0.9, OT-1.1, RD-0.5, RD -0.7, RD -0.9, and RD -1.1.

Taking into account quantitative assessment, physical parameters of porous structure, such as pore size, porosity, volume, surface area, and specific surface area, were paid close attention, which would affect culture conditions and cell differentiation for BTE directly. In the SolidWorks software, pore size could be measured manually, and surface area and volume of porous scaffolds were computed automatically with the analyzing tools, respectively. Additionally, according to (1), porosity* p* could be calculated.

(1) $$\begin{array}{c} p=\frac{V_{0}-V}{V_{0}}\times 100\% \end{array}$$

where *V*_0_ is the volume of initial solid implant;* V* is the volume of porous implant. Among them, *V*_0_ can be calculated according to the volume formula of cube and the known dimension size ( 20mm x 10 mm).

In the meantime, specific surface area* s* could be calculated as

(2) $$\begin{array}{c} s=\frac{V}{S^{*}} \end{array}$$

where* V* and *S*^*∗*^ are the volume and surface area of porous implant, respectively.

### 2.2. Computational Fluid Dynamics

**Figure 2:**
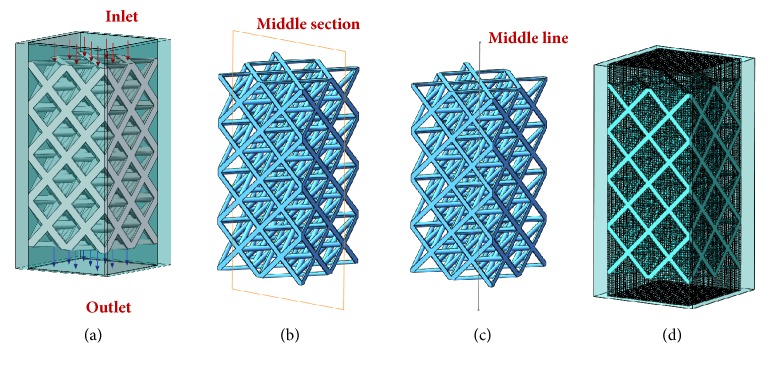
Schematic diagrams of CFD models: (a) boundary condition; (b) middle section view; (c) middle line view; (d) meshing model.

Based on the control design, flow performance of porous scaffolds with two shapes of unit cells and four strut sizes (OT-0.5, OT-0.7, OT-0.9, OT-1.1, RD-0.5, RD -0.7, RD -0.9, and RD -1.1) was evaluated by numerical simulation method in the plugin of SolidWorks software. As shown in Figure 2(a), all of the porous scaffolds were limited in the same enclosed cuboid, respectively, and one side of the cuboid was assumed as the inlet, whilst the opposite side was assumed as the outlet. Dulbecco's modified Eagle's medium (DMEM) was represented as fluid material to simulate a steady state in vitro, whose viscosity and density are 1.45Pa·s and 1000 kg/m^3^ [12].

The boundary conditions defined an inlet velocity (v_i_ = 1 mm/s) at the inlet-flow side and an output pressure (one Atm pressure) at the opposite-flow side. The governing equation underlying the calculation was the Navier-Stokes formulation. Meanwhile, in order to assess flow performances of porous scaffolds with different strut sizes and pore shapes, flow trajectory, flow velocity, and flow shear stress were planned to be obtained and compared. In addition, according to Darcy's law,

(3) $$\begin{array}{c} Q=vA=k\mu_{d}A\frac{\Delta P}{L} \end{array}$$

so the average permeability could be expressed as follows [32]:

(4) $$\begin{array}{c} k=v\mu_{d}\frac{L}{\Delta P} \end{array}$$

where *Q* is the volumetric flow rate;* A* is the area of cross section for pores;* v* is the average velocity; *μ*_*d*_ is the fluid viscosity of DMEM;* L* is the length of the model; Δ*P* is the pressure drop between the inlet and the outlet. Among them,* v* could be computed directly by the flow simulation; Δ*P* could be got by the inlet and output surface probing; *μ*_*d*_ and* L* are known. Then, average permeability* k* could be calculated, respectively, according to (4). Moreover, middle section view (Figure 2(b)) and middle line view (Figure 2(c)) of porous scaffolds were used to display and assess inner flow velocity and flow shear stress in the software, and the cut planes for all the porous scaffolds are at the same position, and they all shared one same midline.

Finally, tetrahedron was used for the models' meshing in the flow simulation (Figure 2(d)), and adaptive optimization function is performed. Furthermore, in order to provide accurate computation and reliable results, convergence studies were conducted to evaluate mesh size for the models before the flow simulation [33]. When the model, boundary conditions, and fluid material were kept the same, velocity and shear stress were probed with different amounts of tetrahedral elements. Then, the results of the convergence study were utilized to choose appropriate analysis accuracy as well as calculating time, and all of the numbers of tetrahedral elements of the meshes are more than 154512 ( Table 1), which could provide enough accuracy.

## 3. Results

### 3.1. Parametric Characterization of Design

**Figure 3:**
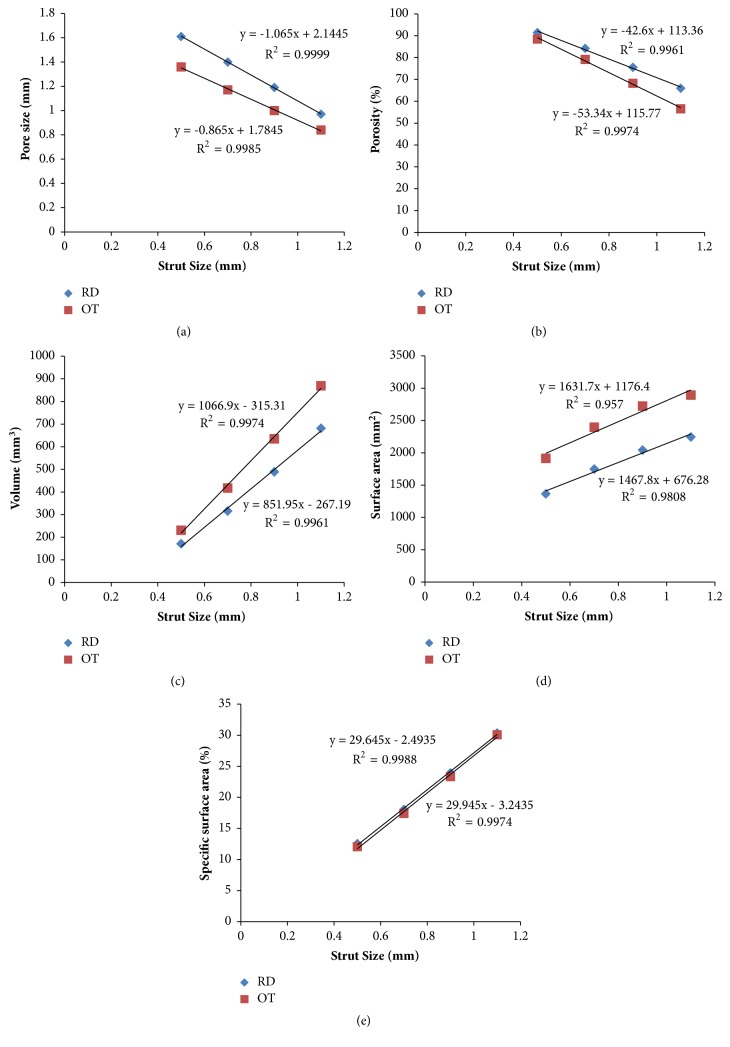
Relationship between strut size and other physical parameters.

As shown in Table 2, under the conditions of the same unit cell size and model size, porous scaffolds with different strut sizes (*φ*0.5, 0.7, 0.9, and 1.1) provided different pore size, porosity, volume, surface area, and specific surface area. Furthermore, for the two shapes, the related parameters showed the same trend: with the increase of strut size, pore size and porosity linearly decrease, but the volume, surface area, and specific surface area increased (Figure 3). Meanwhile, RD scaffolds displayed bigger pore size and porosity than OT scaffolds on the same strut size, but for the volume, surface area, and specific surface area, OT scaffolds seemed to be bigger than RD.

### 3.2. Fluid Flow Performance

#### 3.2.1. Flow Velocity

**Figure 4:**
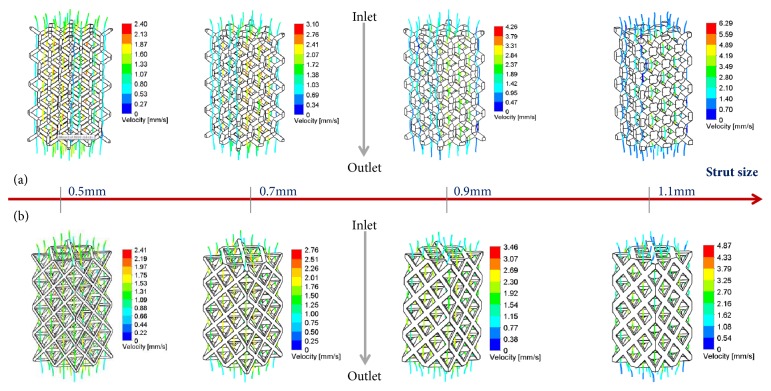
Total flow trajectory and velocity distribution inner porous scaffolds: (a) OT shape; (b) RD shape.

**Figure 5:**
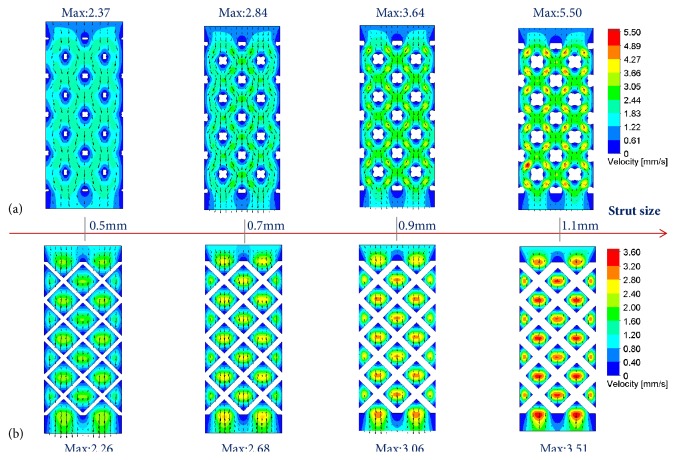
Flow velocity distribution on the middle section view: (a) RD shape; (b) OT shape.

Figure 4 showed the total flow trajectory and velocity distribution of porous scaffolds with different strut sizes, in which maximum velocity magnitudes observed in each shape of unit cell ranged from 2.40 mm/s to 6.29 mm/s for RD shape and 2.41 mm/s to 4.87 mm/s for OT shape. Furthermore, RD-1.1 and OT-1.1 displayed the highest maximum velocity, respectively, but the opposite was displayed for RD-0.5 and OT-0.5. Meanwhile, as illustrated in Figure 5, the maximum velocity on the middle section seemed to increase with the increasing of strut size, and RD scaffolds and OT scaffolds with different strut sizes displayed similar flow trajectory, respectively.

**Figure 6:**
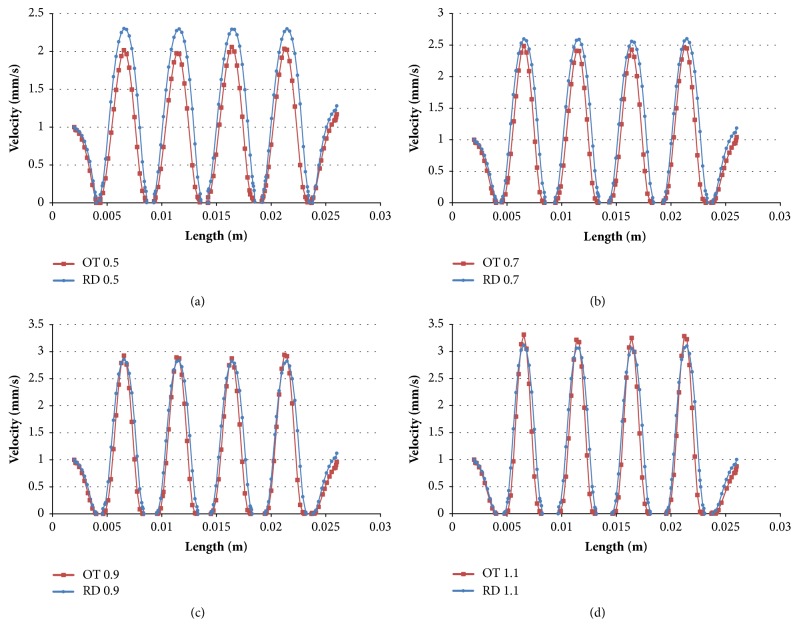
Flow velocity trends of the porous scaffolds with different strut sizes and shapes on the middle line: (a) 0.5 mm; (b) 0.7 mm; (c)0.9 mm; (d)1.1 mm.

In addition, Figure 6 showed the velocity on the middle line, in which RD and OT shapes displayed similar change trends but different amplitudes. For the strut sizes of 0.5mm and 0.7mm, RD scaffolds had bigger amplitude than OT scaffolds (Figures 6(a) and 6(b)), but for the strut sizes of 0.9mm and 0.9mm, OT shapes showed the bigger maximum values (Figures 6(c) and 6(d)). Furthermore, the maximum velocity also increased with the increasing of strut size whether it was RD shape or OT shape, which is similar to the trends of total velocity and middle section velocity.

#### 3.2.2. Flow Permeability

**Figure 7:**
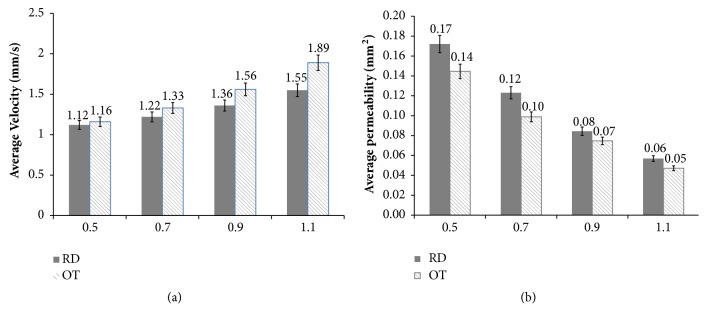
Average velocity and permeability of porous scaffolds: (a) velocity; (b) permeability.

**Figure 8:**
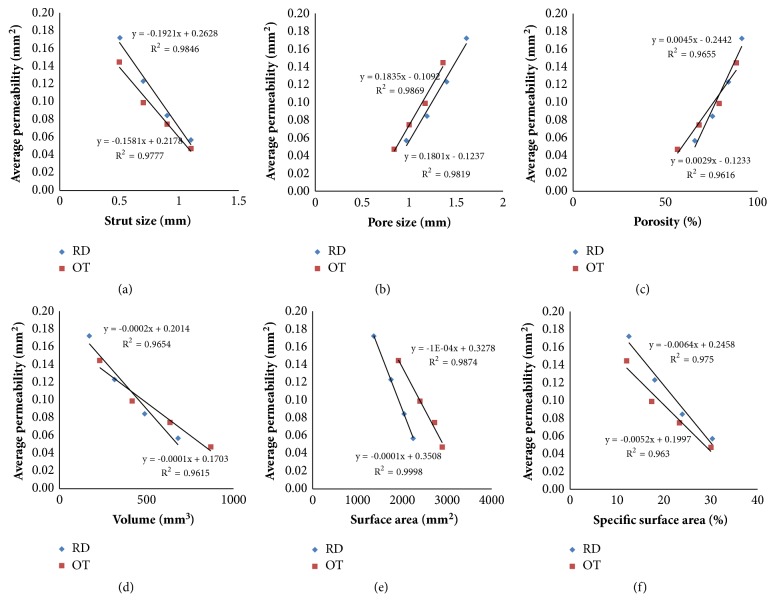
Relationship between average permeability and physical parameters.

As illustrated in Figure 7, with the increasing of strut size, average permeability showed a decreasing trend for RD and OT shapes, but average velocity would increase. Moreover, Figure 8 showed the good correlation observed between average permeability and strut size, pore size, porosity, volume, surface area, and specific surface area. With the increasing of pore size and porosity, the average permeability should be increased, but it would decrease with the volume and surface area increasing.

#### 3.2.3. Flow Shear Stress

**Figure 9:**
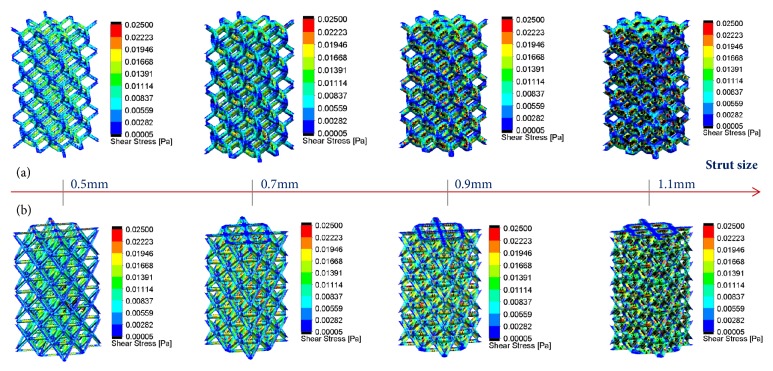
Cloud chart of flow shear stress: (a) RD; (b) OT.

**Figure 10:**
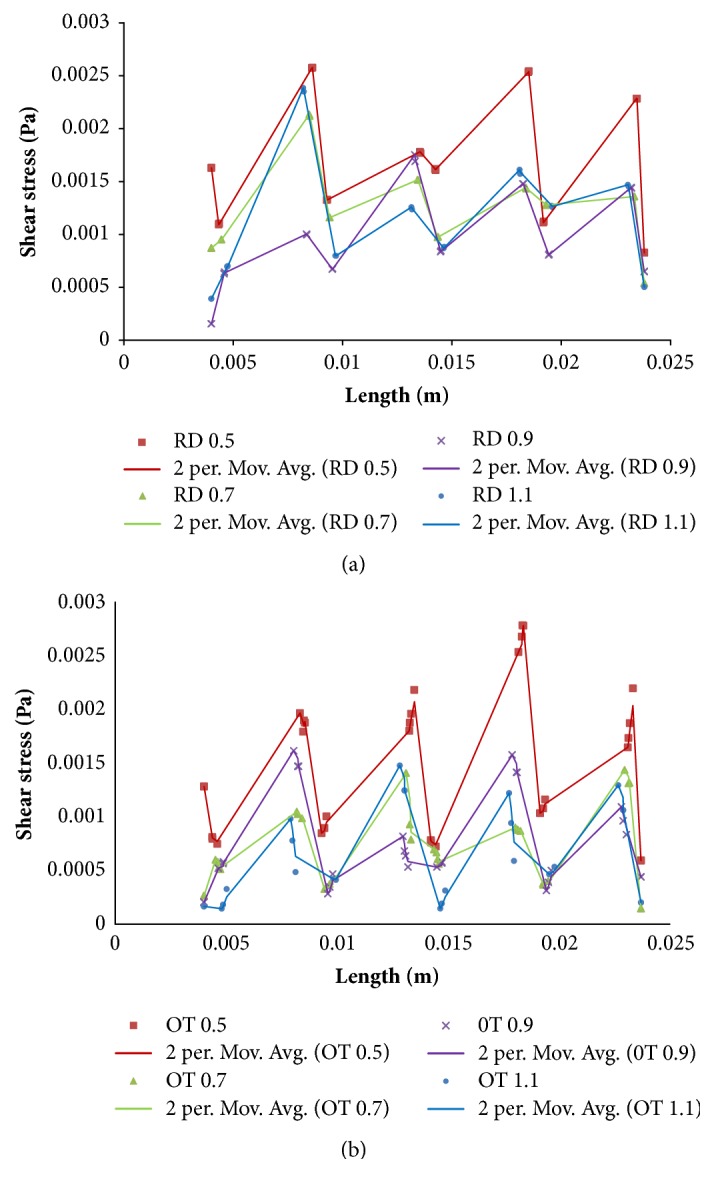
Shear stresses and the change trends along the middle line for the porous scaffolds: (a) RD; (b) OT.

The shear stress distributions on the surface of porous structures were shown in Figure 9. Taking into account previous study [16–19], the cloud chart was limited to from 0.05 to 25 mPa. The black color marked in Figure 9 means that the shear stress was not in the range (>25 mPa or <0.05 mPa), and the red color marked suggested a bigger value. Similarly, the shear stress observed would increase with the strut size, because more red colors have been displayed. Besides, more black colors appeared with the increasing of strut size. In the meantime, Figure 10 showed shear stress distribution and its change trend along the middle line for the porous scaffold.

## 4. Discussion

This study confirmed that different unit cells and strut size result in different physical parameters [4], thus unequal flow performance of porous scaffolds for nutrient delivery and tissue regeneration [34]. This should provide a quantified method for the porous implant's control design and numerical evaluation [35]. Some effect of control design on mass transfer and shear stress could be observed and summarized in this paper.

As shown in Figure 3, pore size, porosity, volume, surface area, specific surface area, and other physical parameters would change with the change of strut size. Specifically, it seems that, with the increase of strut size, pore size and porosity would linearly decrease, but the volume and surface area would increase for the same pore shape. Moreover, whether it is RD or OT, a similar trend was shown. Meanwhile, on the same strut size, RD always displayed bigger pore size and porosity and smaller volume, surface area, and specific surface area than OT. This finding confirmed that the strut size and pore shape play vital role in the CAD-control design for the porous implant with regular unit cell, which is quite consistent with Andy L. Olivares [12] and Yuhang Chen [35]. Consequently, it is feasible to design and tailor suitable pore size, porosity, volume, and surface area only by adjusting unit cell shape and strut size. Further on, the calculation formula in Figure 3 could give great help for the specific purposes in clinical design, such as aimed pore size or porosity. Although some researchers found that pore size above 300 *μ*m benefited the increase of bone formation through vascularization, and pore sizes of 100–400 *μ*m may be good for osteoconduction [36, 37], there is not a way through which the pore size can be designed accurately and directly.

In addition, importantly, different physical parameters could result in different flow performance, which would affect porous scaffold's biological properties [38]. In this study, Figures 4 and 5 showed that same pore shape could display similar inner structures and flow trajectory, but different flow velocity because of different physical parameters. With the increasing of strut size, max-flow velocity would increase. This is quite following theoretical formula (3). The area of cross section* A* would become smaller when the strut size becomes bigger. So the flow velocity would become bigger. However, this study found that different pore shape could result in different change magnitudes for the velocity (Figure 6). For the strut sizes of 0.5 and 0.7mm, RD scaffolds showed bigger amplitude than OT (Figures 6(a) and 6(b)), but for the strut sizes of 0.7 and 0.9mm, OT shapes showed the bigger maximum values (Figures 6(c) and 6(d)) oppositely. It should be noted that pore shape may be the only cause for these results. This phenomenon demonstrates the importance of pore morphology once again.

Compared with flow velocity, flow permeability is an intuitive indicator to evaluate the properties of mass transfer. Previous studies proved that permeability could impact the transport of nutrients and waste products directly, and cells on growth are highly dependent on the nutrients and waste product transfer through the porous structure [39, 40]. In this sense, the study confirmed that different porous structure should lead to different permeability directly. As shown in Figure 7, in contrast with the average flow velocity, average flow permeability of porous scaffolds will decrease with the increasing of strut size, and RD shape always displayed higher permeability than OT on the same strut size. In the meantime, as expected, higher permeabilities were recorded for larger pore sizes and porosity (Figure 8). These results are quite consistent with S. Gómez' study [32], and previous numerical calculations also had confirmed experimental results which indicated that permeability increased with increasing pore sizes and porosity [41]. However, with the increasing of volume, surface area, and specific surface area, average permeability seemed to be decreased. Similarly, linear calculation formulas in Figure 6 also could be used for permeability predictions of OT and RD porous structures.

Furthermore, permeability also impacted cell proliferation and activity by affecting the efficiency of shear stimuli to cells under perfused culture, and proper shear stress from fluid flow has been shown to be favorable for bone regeneration in vitro [35, 42]. For these reasons, the predictions of flow shear stress on regular architecture through computational fluid dynamic can be determinant on the design parameters. This study confirmed that flow shear stress would increase with increasing of strut size, and higher pore sizes and porosity (RD-0.5 and OT-0.5) would provide a better surrounding for tissue regeneration (Figure 9) in the views of Cartmell [17] and Raimondi [18, 19]. Besides, according to Jie Fan's study, higher permeable scaffolds exhibited superior performances and advantages during bone regeneration in vitro. With comprehensive considerations of the results of Figures 7 and 9, the findings in this study are reliable, whether it is from the perspective of shear stress or permeability. Further, this study also found that OT-0.7, OT-0.9, OT-1.1, RD -0.7, RD -0.9, and RD -1.1 displayed increasing shear stress and inappropriate values with the reduction of porosity (Figures 9 and 10), which also proved that bigger porosity could be beneficial to cell adhesion, viability, and proliferation [5–7]. Meanwhile, it could be deduced and proved that porous implant is superior to solid implant because of more appropriate shear stress.

Overall, similar to finite element analysis (FEA) which could evaluate the mechanical properties of the implant [43, 44], CFD is also a reliable and fast method for the flow fluid evaluation, and previous studies had confirmed that flow performance evaluation of porous implant could reflect the biological properties indirectly. For example, L. Olivares et al. studied the interactions between scaffold pore morphology, mechanical stimuli developed at the cell microscopic level, and culture conditions applied at the macroscopic scale on two regular scaffold structures [12]. Ardiyansyah Syahrom et al. simulated idealized cancellous bones from the morphological indices of natural cancellous bone, and permeability of artificial and natural cancellous bone structures was studied [33]. H. Montazerian et al. introduced the radial flow direction as another important factor for scaffold internal architecture design [45]. S. Gómez computed fluid mass transport (permeability) properties and mechanical (elastic) modulus for bone-like implant [32]. Therefore, the effectiveness of the presented method in this study could be accepted. Meanwhile, the findings in this study that the smallest strut size brings higher porosity, permeability, and more appropriate shear stress were partly involved in the literatures [12, 32, 33, 35, 45, 46]. Thus, validation of this study was confirmed, whether it is from the method or from the research results. In addition, in practice, identical total porosity can be designed with quite different pore shape, and identical pore shape also can be designed with different porosity for one same implant, which could lead to different permeability and shear stress, thus resulting in different biological performance for porous implant [20–25]. This study gives a good proof for the abovementioned and could be used to favor mass transfer, and cell adhesion, migration, and new bone apposition (i.e., osteoconduction). Once the virtual models with different unit cells and physical parameters are fully characterized and optimized, unit cell could be rationally selected, and corresponding porous implant could be easily designed and printed by AM for clinical use [26, 27]. Further on, with respect to experience design and traditional implants, the findings in the study would provide a way to quantitatively tailor the porous implant design with optimal biological performance and exhibit a considerable potential in the fabrication of new prosthetic cellular materials for bioengineering applications [35], which has important clinical implications [20, 26, 27, 46].

Finally, although valuable discoveries are revealed by this study, there are also some limitations. On the one hand, only two shapes of unit cells with four strut sizes were simulated in the study, which is not enough to meet various needs in practice. In the future, flow performance of porous implant with more shapes of unit cells and other strut sizes should be studied and focused on in vitro and in vivo in order to create a unit cell library, which is conducive to actual design. On the other hand, it should be noted that this study has been examined only in the view of design [46]. The additive manufacturing process and postprocessing method may also affect mass transfer and tissue regeneration, such as residual powder. Therefore, future research should consider actual manufacturing.

## 5. Conclusion

This study demonstrated that control design with unit cell's shape and strut size is a feasible method to provide appropriate porous implant with better performance of nutrient delivery and cell attachment, viability, and proliferation directly. The results have shown that, with the increasing of strut size, pore size, porosity, and permeability displayed decreasing trend, but volume, surface area, specific surface area, average velocity, and shear stress seemed to increase. Meanwhile, bigger pore size and porosity could bring higher average permeability and more appropriate shear stress, but smaller volume and surface area. Moreover, the findings of how unit cell shape and strut size affect flow velocity, permeability, and shear stress in this study can help implant optimize both nutrient delivery and tissue regeneration, and the formulas in Figures 2 and 6 are meaningful for the quantitative design and analysis.

## Figures and Tables

**Table 1:** Number of tetrahedral elements of the meshes for models.

**Unit cell**	**Scaffolds**	**Cells**	**Fluid cells**	**Solid cells**	**Partial cells**
**OT**	OT-0.5	160000	110841	390	48769
	OT-0.7	160000	98483	4239	57278
	OT-0.9	158740	85625	10212	62903
	OT-1.1	155079	73492	16569	65018

**RD**	RD-0.5	159776	105421	840	53515
	RD -0.7	159482	90263	6888	62331
	RD -0.9	158257	78765	15290	64202
	RD -1.1	154512	58703	24523	71286

**Table 2:** Parametric characterization of porous implant.

**Unit cell**	**Unit cell size (mm)**	**Scaffold models**	**Model size (mm)**	**Strut size (mm)**	**Pore size (mm)**	**Porosity (**%**)**	**Volume (mm** ^**3**^ **)**	**Surface area (mm** ^**2**^ **)**	**Specific surface area (**%**)**
**OT**	5×5×5	OT-0.5	10×10×20	0.5	1.36	88.46	230.9	1914.9	12.06
		OT-0.7		0.7	1.17	79.14	417.2	2395.9	17.41
		OT-0.9		0.9	1.00	68.25	635.1	2722.3	23.33
		OT-1.1		1.1	0.84	56.53	869.5	2893.9	30.05

**RD**	5×5×5	RD-0.5	10×10×20	0.5	1.61	91.44	171.2	1364.3	12.55
		RD-0.7		0.7	1.40	84.21	315.8	1749.5	18.05
		RD-0.9		0.9	1.19	75.54	489.1	2043.2	23.94
		RD-1.1		1.1	0.97	65.93	681.4	2244.9	30.35

## Data Availability

The figures and tables data used to support the findings of this study are included within the article.
